# Metabolic characteristics of different phenotypes in reproductive-aged women with polycystic ovary syndrome

**DOI:** 10.3389/fendo.2024.1370578

**Published:** 2024-07-23

**Authors:** Xinling Wen, Li Wang, E. Bai

**Affiliations:** ^1^ Department of Anesthesiology and Operation, The First Affiliated Hospital of Xi’an Jiaotong University, Xi’an, Shaanxi, China; ^2^ Department of Gynecology and Obstetrics, The First Affiliated Hospital of Xi’an Jiaotong University, Xi’an, Shaanxi, China

**Keywords:** metabolic characteristics, different phenotypes, polycystic ovary syndrome, hyperandrogenism, insulin resistance, metabolic syndrome

## Abstract

**Objective:**

Polycystic ovary syndrome (PCOS) is an endocrine metabolic disorder in reproductive-aged women. The study was designed to investigate the metabolic characteristics of different phenotypes in women with PCOS of reproductive age.

**Methods:**

A total of 442 women with PCOS were recruited in this cross-sectional study. According to different phenotypes, all women were divided into three groups: the chronic ovulatory dysfunction and hyperandrogenism group (OD-HA group, *n* = 138), the chronic ovulatory dysfunction and polycystic ovarian morphology group (OD-PCOM group, *n* = 161), and the hyperandrogenism and polycystic ovarian morphology group (HA-PCOM group, *n* = 143). The metabolic risk factors and prevalence rates of metabolic disorders among the three groups were compared.

**Results:**

The body mass index (BMI), waist circumference, and waist-to-hip ratio (WHR) of women from the OD-HA group and HA-PCOM group were significantly higher than those of women from the OD-PCOM group (*p* < 0.05). The serum insulin concentration and homeostasis model assessment of insulin resistance (HOMA IR) at 2 h and 3 h after oral glucose powder in women from the OD-HA group and HA-PCOM group were significantly higher than those from the OD-PCOM group (*p* < 0.05). The serum total cholesterol (TC), triglyceride (TG), and low-density lipoprotein cholesterol (LDL-C) in women from the OD-HA group and HA-PCOM group were significantly higher than those in women from the OD-PCOM group (*p* < 0.05). The prevalence rates of impaired glucose tolerance (IGT), type 2 diabetes mellitus (T2DM), insulin resistance (IR), metabolic syndrome (MS), nonalcoholic fatty liver disease (NAFLD), and dyslipidemia of women with PCOS were 17.9%, 3.6%, 58.4%, 29.4%, 46.6%, and 43.4%, respectively. The prevalence rates of IGT, IR, MS, NAFLD, and dyslipidemia of women in the OD-HA group and HA-PCOM group were significantly higher than those of women in the OD-PCOM group (*p* < 0.05). T concentration (>1.67 nmol/L) and Ferriman–Gallwey (F–G) score (>3) significantly increased the risk of metabolic disorders in women with PCOS (*p* < 0.05).

**Conclusion:**

The phenotypes of OD-HA and HA-PCOM in women with PCOS were vulnerable to metabolic disorders compared to OD-PCOM. Thus, the metabolic disorders in women with PCOS especially those with the HA phenotype should be paid more attention in order to reduce long-term complications.

## Introduction

Polycystic ovary syndrome (PCOS) is a lifelong metabolic disorder that will likely influence women’s health from adolescence to after menopause (1). The epidemiological investigations showed that the incidence of PCOS in women of reproductive age is 5%–15% according to different diagnostic criteria (2, 3). Although the exact etiology and pathogenesis of PCOS are unclear up to now, its effects on the health of women are well known. Women with PCOS have adverse effects on their health in pregnancy, and the disease also affects the health of their offspring. PCOS is an important risk factor in the development of gestational diabetes mellitus (4). A recent study by Risal et al. reported that daughters of mothers with PCOS have a fivefold increased risk for PCOS (5). Another recent systematic review and meta-analysis by Gunning et al. showed that normal weight children of women with PCOS were prone to developing cardiovascular metabolic disorders in early childhood (6). Furthermore, studies have proven that PCOS is associated with insulin resistance (IR), hyperinsulinemia (HI), type 2 diabetes mellitus (T2DM), metabolic syndrome (MS), and an increased risk of endometrial carcinoma, even in those with normal weight (7, 8). Additionally, women with PCOS are prone to atherogenic dyslipidemia, nonalcoholic fatty liver disease (NAFLD), nonalcoholic steatohepatitis, and increased risk factors for cardiovascular disease (9–11).

The diagnostic criteria have been proposed by different organizations over the past several decades. In 1990, the diagnostic criteria were carried out at a National Institutes of Health (NIH) conference, which required the combination of chronic oligo/anovulation and clinical or biochemical evidence of hyperandrogenism (HA), after the exclusion of related disease (12). Subsequently, the Rotterdam European Society of Human Reproduction and the Embryology/American Society for Reproduction Medicine (ESHRE/ASRM) Consensus Workshop group proposed the addition of ultrasound characteristics for polycystic ovarian morphology (PCOM) to the NIH criteria in 2003, with a statement that women with any two of these three criteria could be diagnosed with PCOS (13). The diagnostic criteria expanded the diagnosis of PCOS, and broadened the complexity of PCOS phenotypes compared with the NIH definition, and resulted in an increased number of women with PCOS. Afterwards, the Androgen Excess Society (AES) proposed new diagnostic criteria and declared that HA is the necessary condition for diagnosis of PCOS (14). However, the Rotterdam criterion is the most widely used diagnostic criteria until now.

PCOS is a heterogeneous disease with diverse clinical manifestations, including menstrual irregularities, HA, infertility, and metabolic disorders. Different reports on the prevalence rate of metabolic disorders and its related long-term implications in women with PCOS are due to different diagnostic criteria. Therefore, this study aims to investigate the metabolic characteristics of different phenotypes in women with PCOS of reproductive age.

## Materials and methods

### Study design and participants

**Figure 1:**
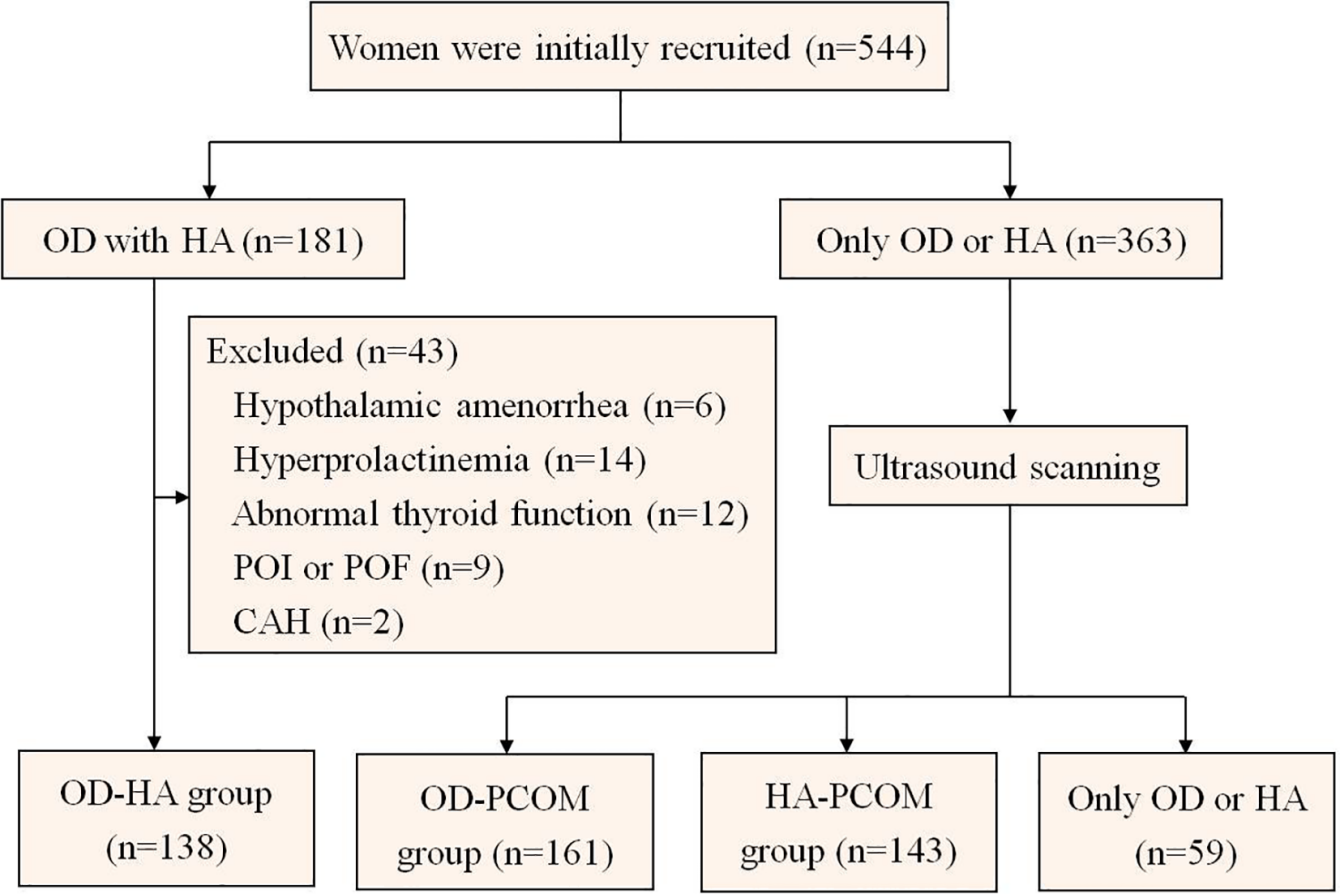
Flowchart of study participation. (OD, ovulatory dysfunction; HA, hyperandrogenism; POI, premature ovarian insufficiency; POF, premature ovarian failure; CAH, congenital adrenal hyperplasia).

**Figure 1** indicates the flowchart of study participation. A total of 544 women in the First Affiliated Hospital of Xi’an Jiaotong University from January 2018 to March 2022 were initially recruited in this cross-sectional study. Among them, 181 women presented chronic ovulatory dysfunction (OD) and HA, and 43 women were excluded due to hypothalamic amenorrhea, hyperprolactinemia, abnormal thyroid function, premature ovarian insufficiency (POI), premature ovarian failure (POF), or congenital adrenal hyperplasia (CAH). In the end, 138 women were selected as part of the OD-HA group. At the same time, ultrasound scanning was performed to detect PCOM among 363 women with only OD or HA; 161 women were selected as part of the OD-PCOM group, while 143 women were selected as part of the HA-PCOM group. A total of 59 women were excluded because they only have OD or HA. The diagnostic criteria of PCOS in this study were the Rotterdam criteria: (a) chronic OD, (b) clinical manifestations or biochemical evidence of HA, and (c) PCOM: the presence of at least 12 antral follicles measuring 2–9 mm in diameter in unilateral ovary or bilateral ovaries, and (or) an increased ovarian volume (≥10 mL). PCOS could be diagnosed when any two of these three criteria were presented (13). Laboratory evidence was defined as an abnormally increased testosterone level (>1.67 nmol/L). Hirsutism was regarded as the clinical manifestations of HA, which was defined as a modified Ferriman–Gallwey score of more than 3 at the time of physical examination. Participants were excluded if they had autoimmune disease or received treatment with hormone drugs in the past 6 months. In addition, participants who received drugs to treat metabolic disorders and HA were also excluded from this study, such as metformin, insulin, statins, combined oral contraceptive, and spironolactone. All participants gave written informed consent according to procedures granted by the Ethics Committee of The First Affiliated Hospital of Medical College of Xi’an Jiaotong University.

Sample size was calculated considering the prevalence rates of metabolic disorders in women with PCOS. According to the method reported in previous literatures, sample size was calculated with the following parameters: probability of type 1 error (α) of 0.05 and type 2 error (β) of 0.20 (power = 80%), the difference between two means to be detected was 0.52, and expected background standard deviation was 1. Based on this, 120 participants were needed in each group. Considering a loss to follow-up rate of 5%–10%, each group eventually needed at least 132 participants.

### Detection of indicators

The basal concentrations of serum sex hormone on 2–4 days of menses, and anti-Müllerian hormone (AMH) concentration were tested in the clinical laboratory of our hospital using the chemiluminescence method. Women took the oral glucose tolerance test (OGTT) and insulin release test (IRT) after fasting for 12 h. Venous blood from the elbow was extracted in the morning on an empty stomach and 1 h, 2 h, and 3 h after taking 75 g of glucose powder to determine the blood sugar and insulin concentrations. Homeostasis model assessment of insulin resistance (HOMA IR) was used to evaluate the degree of IR. The calculation method was as follows: HOMA IR = blood sugar (mmol/L) × insulin (mIU/L)/22.5. At the same time, serum lipid concentrations were detected, including total cholesterol (TC), triglyceride (TG), low-density lipoprotein cholesterol (LDL-C), and high-density lipoprotein cholesterol (HDL-C).

### Outcome measures

The primary outcome was the prevalence rates of metabolic disorders, including impaired glucose tolerance (IGT), T2DM, IR, MS, NAFLD, and dyslipidemia. IGT was defined as fasting blood glucose < 7.0 mmol/L and 7.8 mmol/L ≤ blood glucose 2 h after oral glucose powder < 11.1 mmol/L. T2DM was defined as fasting glucose ≥ 7.0 mmol/L or blood glucose 2 h after oral glucose powder ≥ 11.1 mmol/L (15). IR was defined as HOMA-IR > 3.0 (16). The diagnostic criteria for MS in this study were proposed by the Chinese Diabetes Society, which were more suitable for Chinese people (15) (**Table 1**). Dyslipidemia was defined as presenting any one of the following four criteria: TC ≥ 6.2 mmol/L, TG ≥ 2.3 mmol/L, LDL-C ≥ 4.1 mmol/L, and HDL-C < 1.0 mmol/L (17).

**Table 1:** Diagnostic criteria of MS recommended by the Chinese Diabetes Society (the diagnosis of MS must meet at least three out of the following four criteria).

Criteria	Parameters
Waist circumference	≥85 cm for female patients
Hyperglycemia	Fasting glucose ≥ 6.1 mmol/L or glucose ≥ 7.8 mmol/L 2 h after glucose load and (or) diabetes has been diagnosed and treated
Hypertension	Systolic blood pressure ≥ 130 mmHg or diastolic blood pressure ≥ 85 mmHg, and (or) treatment of previously diagnosed hypertension
Hypertriglyceridemia	Fasting TG ≥ 1.70 mmol/L or fasting HDL-C < 1.04 mmol/L

The secondary outcomes were as follows: (a) the results of general metabolic parameters, including blood pressure, body mass index (BMI), waist circumference, hip circumference, and waist-to-hip ratio (WHR); (b) the results of OGTT, IRT, and blood lipid; and (c) the effects of HA on metabolic disorders.

### Statistical analysis

The data in this study were analyzed using SPSS version 20.0. The Kolmogorov–Smirnov test was used to check the normal distribution prior to statistical tests. For normally distributed variables, the continuous variables were presented as mean ± standard deviation and were analyzed by Student’s *t*-test, whereas the Mann–Whitney *U*-test was used to analyze non-normally distributed data. The chi-square test was used to analyze enumeration data, which were expressed as number and percentage (%). To further explore the effect of HA on metabolic disorders in women with PCOS, the multivariate logistic regression analysis was used, and the BMI, waist circumference, and WHR were adjusted. Adjusted odds ratios (OR) with 95% confidence intervals as relative effect estimates were calculated. *p*-values <0.05 were considered statistically significant.

## Results

### Baseline characteristics of the three groups

**Table 2** shows the baseline characteristics of women among the three groups. The Ferriman–Gallwey score and serum testosterone (T) concentration were significantly lower in women from the OD-PCOM group compared with women in the OD-HA group and HA-PCOM group (*p* < 0.05). However, no significant difference was found when comparing other baseline characteristics among the three groups (*p* > 0.05).

**Table 2:** Baseline characteristics of women among the three groups.

Characteristics	OD-HA group (*n* = 138)	OD-PCOM group (*n* = 161)	HA-PCOM group (*n* = 143)	*p-*value ^a^
Age (years)	32.9 ± 8.3	33.7 ± 7.9	34.1 ± 9.5	0.769
Ferriman–Gallwey score	4.9 ± 1.7^★^	2.8 ± 1.0	4.3 ± 1.4^★^	0.034
Marital status				0.872
Single	21 (15.2)	27 (16.8)	25 (17.5)	
Married	117 (84.8)	134 (83.2)	118 (82.5)	
Smoking	10 (7.2)	13 (8.1)	12 (8.4)	0.935
Family history				
Diabetes mellitus	19 (13.8)	25 (15.5)	20 (14.0)	0.893
Hypertension	16 (11.6)	18 (11.2)	17 (11.9)	0.981
Coronary heart disease	14 (10.1)	15 (9.3)	13 (9.1)	0.951
Thyroid disease	13 (9.4)	14 (8.7)	16 (11.2)	0.757
Basal concentration				
FSH (mIU/mL)	7.3 ± 2.0	6.8 ± 1.9	6.5 ± 1.7	0.665
LH (mIU/mL)	14.8 ± 4.3	11.6 ± 2.7	13.6 ± 3.8	0.413
PRL (ng/mL)	13.5 ± 3.6	12.7 ± 3.3	15.7 ± 4.2	0.338
E_2_ (pmol/L)	89.2 ± 17.4	81.3 ± 16.8	95.3 ± 18.4	0.527
P (nmol/L)	0.9 ± 0.5	1.0 ± 0.6	0.8 ± 0.4	0.901
T (nmol/L)	2.4 ± 0.8^★^	1.2 ± 0.5	2.3 ± 0.9^★^	0.042
LH/FSH	2.0 ± 0.5	1.7 ± 0.4	2.2 ± 0.6	0.376
AMH (ng/mL)	3.9 ± 0.8	3.6 ± 0.7	4.1 ± 1.2	0.259
History of drug therapy^▴^				
Antibiotic	20 (14.5)	22 (13.7)	19 (13.3)	0.956
Vitamins	16 (11.6)	19 (11.8)	14 (9.8)	0.834
Sedative-hypnotics	5 (3.6)	7 (4.3)	4 (2.8)	0.770
History of GDM	2 (1.4)	1 (0.6)	2 (1.4)	0.744

a Variance analysis or chi-square test. Data given as mean ± SD or number (%).

^★^Vs. OD-PCOM group. t-test, p < 0.05.

^▴^Drugs used in the last 6 months.

FSH, follicle-stimulating hormone; LH, luteinizing hormone; PRL, prolactin; P, progesterone; E_2_, estradiol; T, testosterone; GDM, gestational diabetes mellitus.

### General metabolic parameters

The data in **Table 3** demonstrate the general metabolic parameters among the three groups. The BMI, waist circumference, and WHR in women from the OD-HA group and HA-PCOM group were significantly higher than those of women in the OD-PCOM group (*p* < 0.05), but the above parameters had no statistical difference between the OD-HA group and HA-PCOM group (*p* > 0.05). Moreover, no significant difference was found when comparing blood pressure and hip circumference among the three groups (*p* > 0.05).

**Table 3:** Comparison of general metabolic parameters among the three groups.

Parameters	OD-HA group (*n* = 138)	OD-PCOM group (*n* = 161)	HA-PCOM group (*n* = 143)	*p-*value ^a^
Blood pressure				
SBP (mmHg)	105.3 ± 13.2	103.6 ± 12.9	101.2 ± 11.7	0.710
DBP (mmHg)	72.4 ± 8.6	71.3 ± 9.7	69.5 ± 8.4	0.698
BMI (kg/m^2^)	25.3 ± 6.5^★^	22.7 ± 5.1	24.9 ± 5.3^★^	0.046
Waist circumference (cm)	83.4 ± 14.9^★^	77.2 ± 15.3	82.5 ± 13.8^★^	0.039
Hip circumference (cm)	95.2 ± 18.3	96.1 ± 19.2	95.7 ± 17.5	0.457
WHR	0.9 ± 0.4^★^	0.8 ± 0.3	0.9 ± 0.4^★^	0.048

a Variance analysis. Data given as mean ± SD.

^★^Vs. OD-PCOM group. t-test, p < 0.05.

SBP, systolic blood pressure; DBP, diastolic blood pressure.

### Oral glucose tolerance test, insulin release, and blood lipid

**Figure 2:**
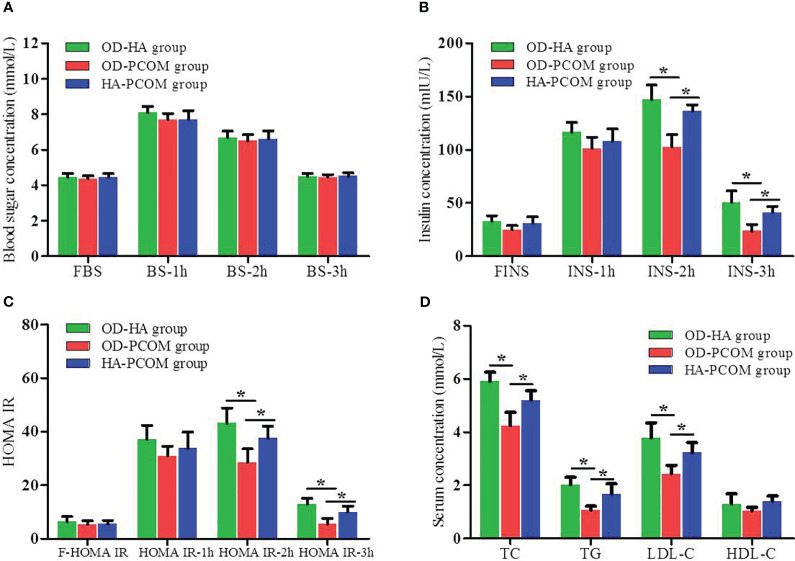
Comparison of the results of OGTT, IRT, and blood lipid among the three groups. **(A)** Blood sugar concentration at different time points after oral glucose powder. **(B)** Insulin concentration at different time points after oral glucose powder. **(C)** HOMA IR at different time points after oral glucose powder. **(D)** Blood lipid. (*p < 0.05).

**Figure 2** reveals the data of OGTT, IRT, and blood lipid among the three groups. The serum insulin concentration and HOMA IR at 2 h and 3 h after oral glucose powder in women from the OD-HA group and HA-PCOM group were significantly higher compared with the OD-PCOM group (*p* < 0.05). Furthermore, the serum TC, TG, and LDL-C in women from the OD-HA group and HA-PCOM group were significantly higher compared with those of the OD-PCOM group (*p* < 0.05). However, no significant difference was found when comparing the above parameters between women in the OD-HA group and HA-PCOM group (*p* > 0.05), and no significant difference was found when comparing blood sugar concentrations among the three groups (*p* > 0.05).

### Prevalence rates of metabolic disorders

**Figure 3:**
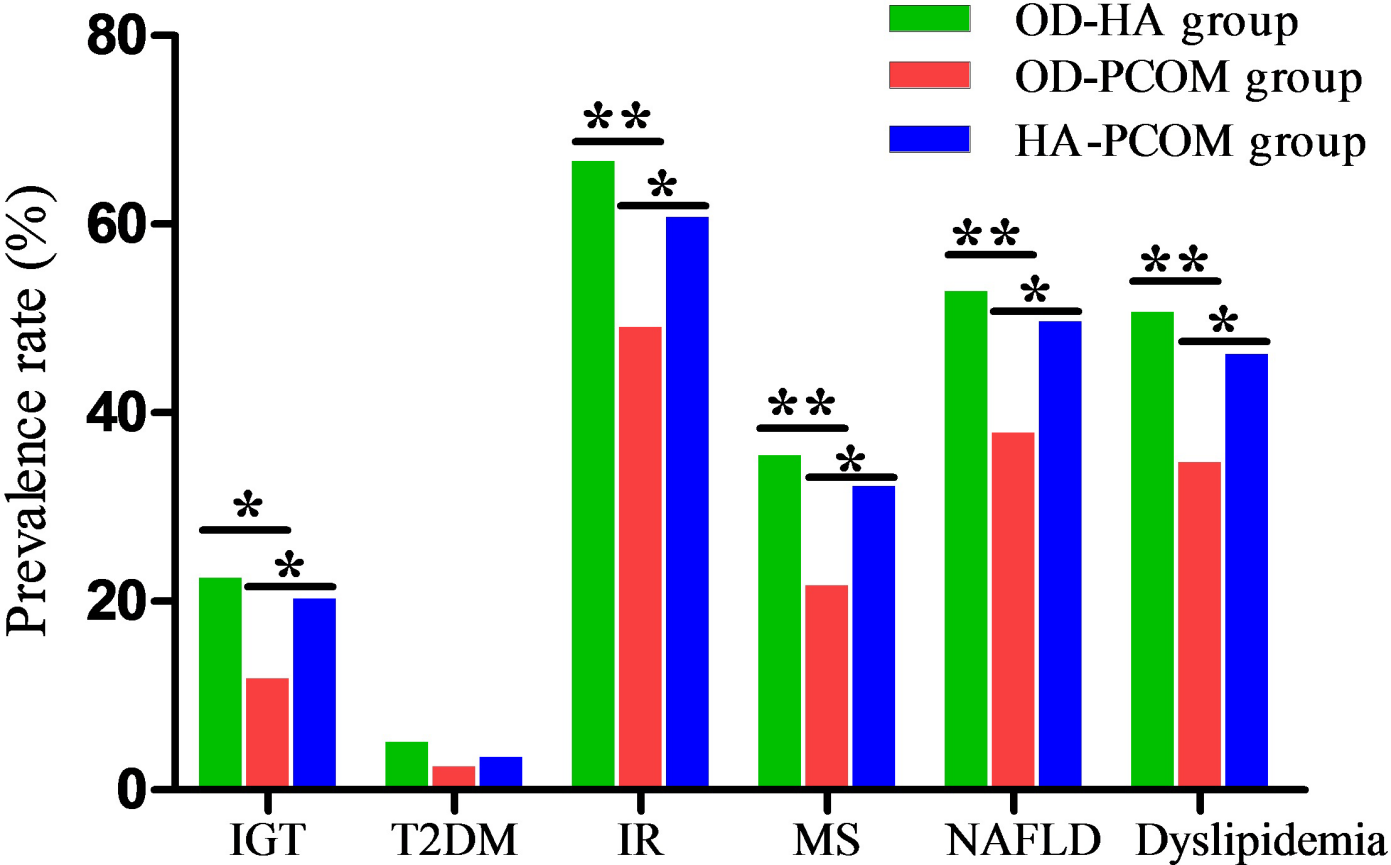
Comparison of prevalence rates of metabolic disorders among the three groups (*p < 0.05, **p < 0.01).

The prevalence rates of IGT, T2DM, IR, MS, NAFLD, and dyslipidemia of women with PCOS were 17.9%, 3.6%, 58.4%, 29.4%, 46.6%, and 43.4%, respectively. The data in **Figure 3** demonstrate the prevalence rates of metabolic disorders among the three groups. The prevalence rates of IGT, IR, MS, NAFLD, and dyslipidemia of women in the OD-HA group and HA-PCOM group were significantly higher compared with women in the OD-PCOM group (22.5% and 20.3% vs. 11.8%, 66.7% and 60.8% vs. 49.1%, 35.5% and 32.2% vs. 21.7%, 52.9% and 49.7% vs. 37.9%, 50.7% and 46.2% vs. 34.8%, respectively) (*p* < 0.05). However, no significant difference was found when comparing the above parameters between women in the OD-HA group and HA-PCOM group (22.5% vs. 20.3%, 66.7% vs. 60.8%, 35.5% vs. 32.2%, 52.9% vs. 49.7%, and 50.7% vs. 46.2%, respectively) (*p* > 0.05). No significant difference was found when comparing the prevalence rate of T2DM among the three groups (5.1%, 2.5%, and 3.5%, respectively) (*p* > 0.05).

### Effect of HA on metabolic disorders

To further explore the effect of HA on metabolic disorders in women with PCOS, the BMI, waist circumference, and WHR were adjusted in the multivariate logistic regression analysis. **Table 4** shows the effect of HA on metabolic disorders. The data demonstrate that HA was an important risk factor in metabolic disorders in women with PCOS. Serum T concentration (>1.67 nmol/L) and F–G score (>3) significantly increased the risk of metabolic disorders (*p* < 0.05).

**Table 4:** Multivariate logistic regression analysis of the effect of HA on metabolic disorders in women with PCOS.

	Standardized β	OR	95% CI	*p*-value
IGT				
T (>1.67 nmol/L)	0.438	1.860	1.233–2.867	0.016
F–G score (>3)	0.204	1.572	1.104–2.139	0.021
IR				
T (>1.67 nmol/L)	0.526	2.009	1.236–3.451	0.012
F–G score (>3)	0.709	2.408	1.309–4.287	0.010
MS				
T (>1.67 nmol/L)	0.319	1.670	1.139–2.402	0.020
F–G score (>3)	0.367	1.803	1.208–2.609	0.018
NAFLD				
T (>1.67 nmol/L)	0.178	1.490	1.100–2.009	0.025
F–G score (>3)	0.210	1.621	1.119–2.233	0.021
Dyslipidemia				
T (>1.67 nmol/L)	0.618	2.013	1.239–3.812	0.011
F –G score (>3)	0.478	1.924	1.244–2.987	0.015

F–G, Ferriman–Gallwey.

## Discussion

PCOS is a kind of heterogeneous disease with metabolic disorders. Studies have confirmed that the interaction among genetic factors, metabolic factors, and environmental factors plays an important role in the pathogenesis of PCOS (18). The clinical manifestations of women with PCOS are disparate in different countries, races, and regions, which show the polymorphism of the disease. HA and IR are important links in the pathogenesis of PCOS, which influence each other and form a vicious cycle (19). Furthermore, IR is also the core pathological mechanism of MS (20). Several studies have shown that most women with PCOS presented as IGT, IR and compensatory HI, abdominal obesity, metabolic disorders, and MS (8, 21). Therefore, on the basis of symptomatic treatment, the long-term complications of women with PCOS should be paid more attention, especially endocrine and metabolic problems.

Our findings indicated that the metabolic characteristics of different phenotypes in reproductive-aged women with PCOS were different. The BMI, waist circumference, and WHR in women from the OD-HA group and HA-PCOM group were significantly higher than those of women from the OD-PCOM group. The serum insulin concentration and HOMA IR of 2 h and 3 h after oral glucose powder in women from the OD-HA group and HA-PCOM group were significantly higher than those from the OD-PCOM group. Furthermore, the serum TC, TG, and LDL-C in women from the OD-HA group and HA-PCOM group were significantly higher than those from the OD-PCOM group. However, no significant difference was found when comparing the above parameters between women in the OD-HA group and HA-PCOM group. In addition, our research also compared the prevalence rates of metabolic disorders among different phenotypes in reproductive-aged women with PCOS. The prevalence rates of metabolic abnormalities were different due to the differences in race, region, lifestyle, age, diagnostic criteria, etc.

Earlier studies of different phenotypes in PCOS displayed that the prevalence of IR and MS in oligomenorrhoeic but normoandrogenemic (PO) women were lower than in PHO women (PCO morphology, hyperandrogenemic, and oligomenorrhoeic), and these results suggested that normoandrogenemic and oligomenorrhoeic women with PCOS are metabolically similar to control women with significantly fewer metabolic features than women with PCOS who are also hyperandrogenemic (22). Data in this study indicated that the prevalence rates of IGT, T2DM, IR, MS, NAFLD, and dyslipidemia of women with PCOS were 17.9%, 3.6%, 58.4%, 29.4%, 46.6%, and 43.4%, respectively. Studies have shown that the prevalence rates of IR and dyslipidemia in women with PCOS were 50–70% and 70%, respectively, and women with PCOS are vulnerable to higher concentrations of TC, LDL-C, and TG (8). The results of investigation from Beijing showed that the prevalence rate of MS in women with PCOS was 31.9% (23). Furthermore, the increased concentrations of TC and TG not only promoted the adverse effect of LDL on metabolism, but also weakened the protective effect of HDL-C on metabolism. Taken together, the above changes in blood lipid could promote the occurrence and development of atherosclerosis and MS. In addition, we also compared the prevalence rates of metabolic disorders among different phenotypes of women with PCOS. Data displayed that the prevalence rates of IGT, IR, MS, NAFLD, and dyslipidemia of women in the OD-HA group and HA-PCOM group were significantly higher than those of women in the OD-PCOM group. However, no significant difference was found when comparing the above parameters between women in the OD-HA group and those in the HA-PCOM group. Moreover, no significant difference was found when comparing the prevalence rate of T2DM among the three groups.

Multivariate regression analysis revealed that HA was an important risk factor in metabolic disorders in women with PCOS. Serum T concentration (>1.67 nmol/L) and F–G score (>3) significantly increased the risk of metabolic disorders. HA plays an important role in metabolic disorders. A number of previous studies have reported that women with PCOS with HA are more prone to metabolic disorders (24). A systematic review in 2020 showed that metabolic disorders in women with PCOS were closely related to HA and IR (25). A study of women with PCOS from the Netherlands found that the occurrence risk of IR and MS increased significantly in women with HA (26). Similarly, Li et al. pointed out that waist circumference combined with free testosterone index (FAI) could be used to predict IR and MS in women with PCOS (27). HA was associated with abnormal fat metabolism. Adipose tissue is the key target of androgen action. Roland et al. reported higher fasting glucose and IGT in prenatally androgenized female mice (28). A recent study reported that the visceral adipose tissue mass of patients with PCOS with HA phenotype was significantly increased, and serum androgen concentration was correlated with IR (29). In brief, the relationship between HA and metabolic disorders is very complex. HA can cause metabolic disorders in several ways, including fat metabolism, glucose-regulating pathways, and islet beta-cell dysfunction (30).

This study still has some limitations. First, the endocrine and metabolic disorders of PCOS are complex. Other androgen metabolic indicators, such as FAI and sex hormone binding globulin, were not detected. Second, some risk factors for metabolic abnormalities were not examined in this study, such as molecular markers of chronic inflammation. Therefore, the accuracy and repeatability of this study need to be proven in the future.

## Conclusion

The metabolic characteristics of different phenotypes in reproductive-aged women with PCOS were different. The PCOS phenotypes of OD-HA and HA-PCOM are vulnerable to metabolic disorders compared to OD-PCOM. HA plays a crucial role in increasing the risk of metabolic disorders in women with PCOS. Targeting HA is likely to become an effective approach in the treatment of PCOS metabolic disorders. However, the metabolic characteristics of PCOS are very complex, and it is not just HA that affects metabolic disorders. In fact, HA and metabolic disorders may influence each other and form a vicious cycle. Therefore, the mechanism and specific relationship between HA and metabolic disorders need to be further studied in the future.

## Data availability statement

The original contributions presented in the study are included in the article/supplementary material. Further inquiries can be directed to the corresponding author.

## Ethics statement

The studies involving humans were approved by The First Affiliated Hospital of Xi’an Jiaotong University Institutional Review Board. The studies were conducted in accordance with the local legislation and institutional requirements. The participants provided their written informed consent to participate in this study.

## Author contributions

XW: Writing – review & editing, Data curation, Investigation. LW: Writing – review & editing, Conceptualization, Writing – original draft. EB: Investigation, Writing – review & editing.
